# Initiation of resuscitation in the delivery room for extremely preterm infants: a profile of neonatal resuscitation instructors

**DOI:** 10.6061/clinics/2016(04)06

**Published:** 2016-04

**Authors:** Cristiane Ribeiro Ambrósio, Adriana Sanudo, Maria Fernanda Branco de Almeida, Ruth Guinsburg

**Affiliations:** I Departamento de Pediatria, Universidade Federal de Uberlândia, Uberlândia/MG, Brazil; II Universidade Federal de São Paulo, Bioestatística, São Paulo/SP, Brazil; III Universidade Federal de São Paulo, (UNIFESP), Pediatria, Neonatologia, São Paulo/SP, Brazil

**Keywords:** Cardiopulmonary Resuscitation, Decision Making, Medical Ethics, Fetal Viability, Extremely Premature Infant

## Abstract

**OBJECTIVE:**

The goal of the present study was to examine the decisions of pediatricians who teach neonatal resuscitation in Brazil, particularly those who start resuscitation in the delivery room for newborns born at 23-26 gestational weeks.

**METHODS:**

The present study was a cross-sectional study that used electronic questionnaires (Dec/11-Sep/13) sent to instructors of the Neonatal Resuscitation Program of the Brazilian Society of Pediatrics. The primary outcome was the gestational age at which the respondent said that he/she would initiate positive pressure ventilation in the delivery room. Latent class analysis was used to identify the major profiles of these instructors, and logistic regression was used to identify variables associated with belonging to one of the derived classes.

**RESULTS:**

Of 685 instructors, 82% agreed to participate. Two latent classes were identified: ‘pro-resuscitation' (instructors with a high probability of performing ventilation on infants born at 23-26 weeks) and ‘pro-limitation' (instructors with a high probability of starting ventilation only for infants born at 25-26 weeks). In the multivariate model, compared with the ‘pro-limitation' class, ‘pro-resuscitation' pediatricians were more likely to be board-certified neonatologists and less likely to base their decision on the probability of the infant's death or on moral/religious considerations.

**CONCLUSION:**

The pediatricians in the most aggressive group were more likely to be specialists in neonatology and to use less subjective criteria to make delivery room decisions.

## INTRODUCTION

The ethical dilemmas associated with neonatal resuscitation in the delivery room are evident in clinical practice. These situations occur when health professionals and/or parents are faced with a choice between two or more conflicting courses of action without clear evidence to support either as the best choice. Dealing with uncertainties and conflicts of various possible courses of action is one of the greatest challenges of neonatal care 1.

In Brazil, the guidelines of the Neonatal Resuscitation Program of the Brazilian Society of Pediatrics (Brazilian NRP) suggest that whenever possible, the resuscitation of extremely preterm infants should be discussed with the pregnant woman and her family before birth. In general, infants with a gestational age of <23 weeks should not be resuscitated due to their limited viability 2.

Although these general recommendations serve as guidance for the work of pediatricians in the delivery room, they do not address the question that determines physicians' attitudes toward the birth of a preterm infant with limited survival potential: what is the best approach to take? Therefore, the factors those physicians consider when deciding whether to initiate resuscitation for extremely premature newborns in the delivery room are unknown. The result may be a discrepancy between existing recommendations and the medical practices that are effectively adopted during daily procedures in the delivery room.

In this context, this study aimed to assess the profiles of pediatricians who teach resuscitation in Brazil with respect to their opinions regarding the resuscitation of extremely preterm infants according to gestational age. We also sought to determine which characteristics of these pediatricians were associated with the initiation or non-initiation of positive pressure ventilation (PPV) in the delivery room for infants born between 23 and 26 weeks of gestation.

## METHODS

After receiving approval from the Research Ethics Committee of the Federal University of São Paulo and the Executive Board of the Brazilian Society of Pediatrics, this cross-sectional study was conducted between December 2011 and September 2013 using an electronic questionnaire that was distributed to all pediatricians who were active instructors of the Brazilian NRP.

An electronic questionnaire was developed based on the questionnaire that was validated and used by Martinez et al. 3. This questionnaire evaluates parental counseling practices, the clinical limits for resuscitation of extremely premature infants, and the medical considerations in decision making for this group of infants to determine the degree to which physicians' beliefs, parents' opinions, and medical resources influence decision-making regarding the resuscitation of newborns at the limit of viability.

Each instructor received the survey link and a personal access password by e-mail. After signing in, each instructor received guidelines about the study and, after agreeing to participate via acceptance of the informed consent form, was granted access to the questionnaire. The questionnaire contained 17 questions and three clinical cases, and answering all items was mandatory. The responses were accessible only to the main researcher, and the instructors were guaranteed anonymity with regard to the survey results.

The analyzed outcome was the respondents' opinion regarding the initiation of resuscitation for preterm newborns at the limit of viability. According the International Liaison Committee on Resuscitation (ILCOR) 4 and the Brazilian NRP 2, after birth, the newborn is placed under a radiant heat source, the head and neck are placed in a slightly extended position, and the mouth and nose are suctioned if excess secretions are present. Newborns with bradycardia or apnea should receive PPV. Consequently, the initiation of resuscitation in the delivery room implies the initiation of PPV using a facemask or a tracheal tube. Therefore, the main outcome of the study was the gestational age at which the respondent reported the intention to perform PPV using either a facemask or tracheal tube.

For the statistical analysis, latent class analysis (LCA) 5 was applied to empirically investigate the pediatricians' response pattern regarding the initiation of PPV in extremely premature infants according to gestational age. To extract the latent classes, fit statistics were applied, including the log-likelihood (LL), Akaike information criterion (AIC) 6, Bayesian information criterion (BIC) 7, sample size-adjusted Bayesian information criterion (SSABIC)^8^, Vuong Lo Mendell Rubin (VLMR) test 9, and an entropy measurement 10. In addition to the indices described, the final evaluation of the best-fit model considered the solution that reflected coherence and revealed conceptually distinct groups. The latent classes were extracted using Mplus version 7.0 11. After calculating the number of latent classes and creating the classes, a multinomial logistic regression model was developed using Stata/SE 13 (Stata Corp LP, College Station, Texas, USA) to evaluate the variables associated with the profile of the latent classes found 12. The final logistic regression model initially included all variables with a *p*-value <0.20 in the univariate analysis, and these variables were removed from the model when the p-value reached >0.05.

## RESULTS

During the study period, there were 685 active instructors in the PRN-SBP, of which 560 (82%) completed the questionnaire. All Brazilian states were represented by at least 75% of their instructors. The general characteristics of the respondents are shown in Table 1.

Five latent class models were examined, ranging from one to five classes. The selected model contained two classes because it not only exhibited the lowest BIC and highest entropy value but also showed adequate values for the remaining fit indices and good interpretability of the event (Table 2).

**Figure 1:**
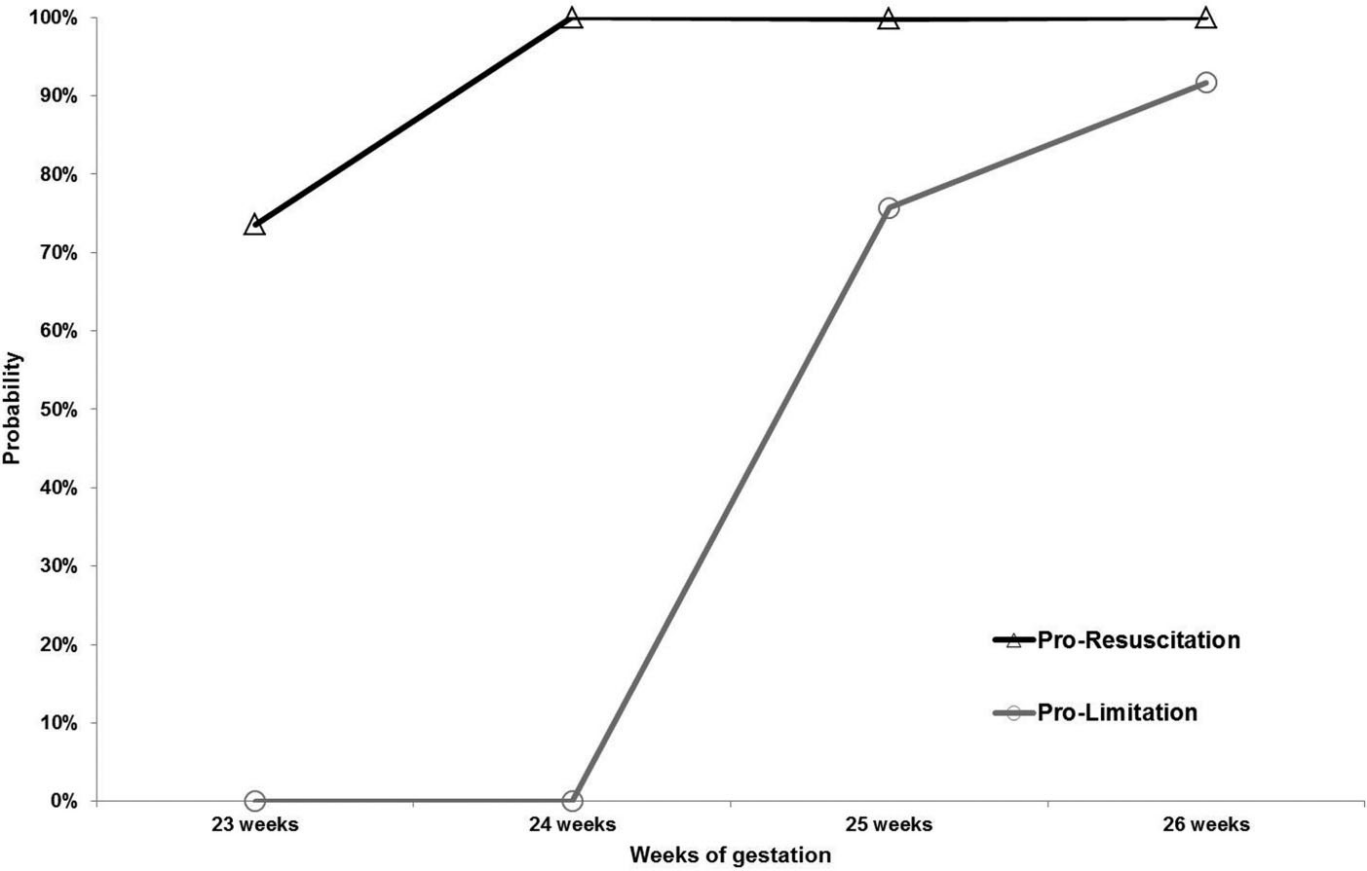
Probability of initiating positive pressure ventilation according to the latent class (‘pro-resuscitation' and ‘pro-limitation') of the survey respondents (N=559) and gestational age.

The classes were designated ‘pro-resuscitation' (which included 81% of the respondents) and ‘pro-limitation' (which included 19% of the respondents). The survey respondents in the ‘pro-resuscitation' class demonstrated a high probability of performing ventilation at all gestational ages. Those in the ‘pro-limitation' class were highly likely to initiate PPV at 25 and 26 weeks of gestation (76% and 92%, respectively), but had a null probability of initiating PPV at 23 and 24 weeks of gestation (Figure 1).

A comparison of the demographic data and occupational characteristics of the respondents of these two classes (Table 3) indicated no significant differences between the two groups. Of the 451 instructors classified as ‘pro-resuscitation', 40 (9%) reported working in units that had written rules about the limits of viability, and 10 (9%) of the 108 instructors in the ‘pro-limitation' class reported the same conditions (*p*=0.872).

The comparison of the criteria used by the two groups to determine the gestational age that resuscitation should be initiated is shown in Table 4. Both groups valued the biological factors of the newborns equally (birth weight and presence of congenital anomalies), but other criteria were valued differently, including the future of the newborn (probability of death, quality of life, and infant suffering), financial issues related to the newborn's family, the institution, and the health care system in general and the value attributed to moral and religious values.

In the final multivariate model, instructors in the ‘pro-resuscitation' group showed an increased likelihood of having a specialist degree in neonatology (OR_adjusted_ 1.605; 95% CI, 1.041–2.474; *p* = 0.032) and a decreased likelihood of using the possibility of infant death (OR_adjusted_ 0.415; 95% CI, 0.260–0.662; *p* < 0.001) and moral/religious considerations (OR_adjusted_ 0.498; 95% CI, 0.305–0.814; *p* = 0.005) as criteria in the decision to initiate resuscitation compared with the ‘pro-limitation' group.

## DISCUSSION

The LCA statistical model selected for this study has been increasingly applied in psychiatry and psychology research. However, its use to determine the profiles of physicians with different medical attitudes regarding ethical dilemmas related to neonatal resuscitation in the delivery room is original. Our study revealed two distinct profiles, including the ‘pro-resuscitation' group, which exhibited a high probability of performing ventilation at all gestational ages, and the ‘pro-limitation' group, which exhibited a high probability of initiating PPV at 25 and 26 weeks and a null probability of initiating PPV at 23 and 24 weeks. The personal characteristics of the instructors did not differ between the two groups.

The two pediatrician profiles identified in this study indicated different viewpoints about the initiation of life support measures in the delivery room and reflected existing doubts about the resuscitation of extremely premature infants at the limit of viability 13-18. A defined group of physicians preferred to provide access to all available technology and assumed the risk of distinct outcomes, including death, pain and suffering, survival with sequelae, and survival without sequelae or with few sequelae. This final possibility seemed to be taken into greater consideration by these pediatricians. It is noteworthy that this group of pediatricians also had more specialized knowledge in neonatology and a higher probability of being board-certified neonatologists. However, another group of physicians preferred to restrict life support measures, particularly considering the possibility of death as an outcome, and considered moral and ethical issues associated with their own practice and those of the newborns' families.

It is possible to divide the factors that influence the opinions of the respondents regarding the initiation of PPV in extremely premature infants into four main areas: biological, future prognosis, ethical and religious issues, and financial issues. The conservative ‘pro-limitation' group considered the combination of all variables when making decisions in the delivery room. Therefore, biological issues, such as the presence of severe congenital anomalies, are considered in decision-making. Similarly, the prognosis, including the probability of death, future quality of life, and infant suffering, is a factor that restricts the measures adopted in the delivery room, in addition to ethical, religious, and financial considerations. In Sweden, a study of active neonatologists indicated that those who considered the future quality of life of newborns were more willing not to perform neonatal resuscitation in extremely premature infants born at the limit of viability 19. In the United States, a study involving all neonatologists in the state of Illinois revealed that religious beliefs did not influence the decision to initiate resuscitation in the delivery room, whereas fear of ethical and professional lawsuits and the ethical discussions during medical training were associated with a lower likelihood of resuscitation for infants with gestational ages closer to the lower limit of viability 20.

The ‘pro-resuscitation' group, in turn, particularly valued the benefits of resuscitation in the delivery room, acknowledging the subjectivity and uncertainty inherent in medical decisions at birth and postponing the decision to limit the therapeutic effort until a later time when more information is available. In contrast to the findings of this study, a previous study by Rebagliato et al. involving 19 European countries 21 found that being Protestant or not valuing religion, having intermediate professional experience (6-15 years), and working in places with large numbers of infants with very low birth weight were associated with a reduced willingness to perform resuscitation in the delivery room. In a similar study involving Lebanese pediatricians and neonatologists, those who worked in level 3 neonatal intensive care units (ICUs) resuscitated significantly more infants with birth weights at the lower limit of viability than did pediatricians who worked in locations without neonatal ICUs 22. Duffy and Reynolds 23 interviewed physicians from 63 neonatal ICUs in southeast England and found that more experienced physicians were more aggressive with respect to resuscitation and more likely to resuscitate infants born at 22 weeks of gestation or less. These findings are similar to those of our study and may indicate that specialization is associated with an increased likelihood of resuscitating preterm infants with a very low gestational age.

Importantly, neither group reported being significantly influenced by the opinions of the infant's parents. In a study by Wiess et al. 20, neonatologists who reported performing resuscitation at the lower limit of viability did not consider the opinions of the newborns' parents. In a survey conducted in 19 European countries, the family was involved in the decision to resuscitate 23-week-old infants in 12 countries. However, when the opinions of the physicians and parents diverged, the final decision was made by the attending physician in 16 of the 19 countries 24. This finding is also consistent with the results of Duffy and Reynolds 22 in the United Kingdom who found that 45% of the physicians would resuscitate a 23-week-old infant who was born healthy or showed visible signs of life at birth, even against the wishes of the parents. According to De Leeuw et al. 25 and Orfali 26, physicians seem to follow the desire of the parents when they request continued treatment, but not when they request limiting or withdrawing treatment. In Brazilian pediatric ICUs, according to Piva et al. 27, the families often lack accurate information regarding the disease and its prognosis; decisions are made unilaterally, and parents generally do not have the opportunity to discuss the therapeutic options to be used with their children.

Finally, it is noteworthy that although financial considerations appear to influence the decision to limit resuscitation in the delivery room, they were not highly valued by either of the two classes of pediatricians evaluated in this study.

The present study has limitations, particularly because the study was based on responses to a questionnaire, which may reflect the intentions of the respondent but may differ from actual medical practice. Another limitation is associated with the selection of a questionnaire with closed answers on a subject in which subtle changes in language may reflect wide variations in medical practice. Despite these limitations, this study indicates that subjective factors directly influence decision-making in the delivery room and that Brazilian pediatricians serving as neonatal resuscitation instructors have different opinions about clinical decisions involving the birth of preterm newborn infants. It is possible to distinguish two groups of physicians who perform practices based on scientific recommendations but have different opinions. Therefore, debates on the topic and generation of accurate information are essential to guide decisions involving extremely premature infants. Furthermore, relevant ethical issues should be addressed to reach a minimum consensus on the care of premature newborns at the limit of viability.

## AUTHOR CONTRIBUTIONS

Ambrosio CR, Sanudo A, Almeida MF and Guinsburg R conceptualized and designed the study, collected the data, carried out all of the analyses and data interpretation, drafted the initial manuscript, reviewed and revised the manuscript, and approved the manuscript final version as submitted.

## Figures and Tables

**Table 1:** Demographic characteristics of the instructors.

	Number	Frequency
Age (years)	44.9±8.7	(27–67)
≥45 years	296	53%
Women	440	78.6%
Married or in a stable relationship	435	77.8%
With children	443	79.8%
Christian	517	92.3%
Consider religion very important	349	62.3%
Hold a specialist degree in pediatrics	467	83.4%
Hold a specialist degree in neonatology	306	54.6%
Neonatology practice for ≥10 years	403	72,7%
Work in the delivery room	490	87.5%
Work in a teaching hospital	243	43.4%
Work in a neonatal ICU	452	80.7%
Experience with end-of-life decisions	226	40.4%

**Table 2:** Comparison of the fit statistics according to the number of latent classes for the outcome ‘initiation of positive pressure ventilation' among the 560 questionnaire respondents.

Model	LL	AIC	BIC	SSABIC	*p-*Vuong	Entropy
1 class	–4,447.72	8,907.44	8,933.41	8,914.36	N.A.	N.A.
2 classes	–4,274.44	8,572.87	8,264.81	8,586.71	<0.001	0.999
3 classes	–4,259.95	8,555.91	8,633.81	8,576.67	<0.001	0.998
4 classes	–4,230.20	8,508.40	8,612.27	8,536.08	<0.001	0.875
5 classes	–4,217.42	8,494.84	8,624.68	8,529.44	0.001	0.841

LL: log-likelihood; AIC: Akaike information criterion; BIC: Bayesian information criterion; SSABIC: sample size-adjusted Bayesian information criterion; p-Vuong: *p*-value associated with the Vuong-Lo-Mendell-Rubin (VLMR) test.

**Table 3:** Demographic characteristics of the respondents according to the latent class (‘pro-resuscitation' or ‘pro-limitation') for the outcome ‘initiate positive pressure ventilation'.

	Latent class	
	Pro-resuscitation	Pro-limitation	*p*-value
Brazilian region*			0.232
Northeast	139 (30.8%)	40 (37.0%)	
North	51 (11.3%)	17 (15.7%)	
Midwest	28 (6.2%)	8 (7.4%)	
South	60 (13.3%)	9 (8.3%)	
Southeast	174 (38.5%)	34 (31.5%)	
**Female**	357 (79.0%)	83 (76.8%)	0.628
Age (years)**	45.1±8.6	43.9±9.0	0.216
**Stable relationship**	353 (78.1%)	82 (75.9%)	0.626
**With children**	362 (80.1%)	85 (78.7%)	0.747
**Christian**	418 (92.5%)	99 (91.7%)	0.776
**Considers religion important**	284 (62.8%)	65 (60.2%)	0.610
**Specialist in neonatology**	256 (56.6%)	50 (46.3%)	0.052
Years of neonatology**	15.7±8.3	15.1±8.4	0.513
**Works in the delivery room**	397 (87.8%)	93 (86.1%)	0.627
**Works in a university hospital**	204 (45.1%)	39 (36.1%)	0.089
**Works in a neonatal ICU**	366 (81.0%)	86 (79.6%)	0.248

* Brazilian region in which the physician practices. **Values are expressed as the means±standard deviations.

**Table 4:** Criteria the respondents considered in the decision to initiate positive pressure ventilation according to the latent classes ‘pro-resuscitation' or ‘pro-regulation'.

	Latent class				
	Pro-resuscitation	Pro-regulation	OR	95% CI	*p-*value
**Birth weight**					0.841
No	209 (46.4%)	49 (45.4%)	1.000		
Yes	241 (53.6%)	59 (54.6%)	0.958	[0.628–1.460]	
**Severe congenital anomaly**								0.061
No	69 (15.3%)	9 (8.3%)	1.000		
Yes	382 (84.7%)	99 (91.7%)	0.503	[0.243–1.043]	
**Possibility of newborn death**								<0.001
No	222 (49.2%)	30 (27.8%)	1.000		
Yes	229 (50.8%)	78 (72.2%)	0.397	[0.251–0.628]	
**Quality of life of the newborn**								0.001
No	203 (45.0%)	29 (26.9%)	1.000		
Yes	248 (55.0%)	79 (73.2%)	0.449	[0.282–0.713]	
**Suffering of the newborn**								0.006
No	234 (51.9%)	40 (37.0%)	1.000		
Yes	217 (48.1%)	68 (63.0%)	0.546	[0.354–0.840]	
**Parents' opinion**								1.000
No	275 (61.1%)	66 (61.1%)	1.000		
Yes	175 (38.9%)	42 (38.9%)	1.000	[0.650–1.538]	
**Moral/religious considerations**								0.001
No	376 (83.4%)	75 (69.4%)	1.000		
Yes	75 (16.6%)	33 (30.6%)	0.453	[0.281–0.732]	
**Cost of the ICU**								0.040
No	442 (98.0%)	102 (94.4%)	1.000		
Yes	9 (2.0%)	6 (5.6%)	0.346	[0.121–0.994]	
**Health resources**								0.004
No	425 (94.2%)	93 (86.1%)	1.000		
Yes	26 (5.8%)	15 (13.9%)	0.379	[0.193–0.744]	
